# Detecting Emerging Diseases in Farm Animals through Clinical Observations

**DOI:** 10.3201/eid1202.050498

**Published:** 2006-02

**Authors:** Gwenaël Vourc'h, Victoria E. Bridges, Jane Gibbens, Brad D. De Groot, Lachlan McIntyre, Roger Poland, Jacques Barnouin

**Affiliations:** * Institut National de la Recherche Agronomique, Theix, France;; † US Department of Agriculture, Fort Collins, Colorado, USA;; ‡ Defra, London, United Kingdom;; § Kansas State University, Manhattan, Kansas, USA;; ¶ EpiCentre, Massey University, Palmerston North, New Zealand;; # Ministry of Agriculture and Forestry, Wellington, New Zealand

**Keywords:** emerging diseases, animal, detection, epidemiology, information systems, perspective

## Abstract

Predicting emerging diseases is among the most difficult challenges facing researchers and health managers. We present available approaches and tools to detect emerging diseases in animals based on clinical observations of farm animals by veterinarians. Three information systems are described and discussed: Veterinary Practitioner Aided Disease Surveillance in New Zealand, the Rapid Syndrome Validation Project—Animal in the United States, and "émergences" in France. These systems are based on syndromic surveillance with the notification of every case or of specific clinical syndromes or on the notification of atypical clinical cases. Data are entered by field veterinarians into forms available through Internet-accessible devices. Beyond challenges of implementing new information systems, minimizing economic and health effects from emerging diseases in animals requires strong synergies across a group of field partners, in research, and in international animal and public health customs and practices.

## Abstract 2

Clinical observations will allow early detection of emerging diseases in animal to enhance response time and capabilities.

After the discovery of antimicrobial drugs, the increased knowledge in pathogenesis, and the improvement of health management, infectious diseases were thought to be a concern restricted to the application of known control measures. However, the dramatic spread of highly pathogenic diseases such as AIDS and multidrug-resistant bacterial infections led the scientific community to seriously examine emerging infectious diseases (*1*). Additionally, most of the emerging issues for humans are zoonotic (*2*) (e.g., avian influenza, bovine spongiform encephalopathy [BSE], severe acute respiratory syndrome [SARS], West Nile virus fever). Consequently, emerging diseases are now being addressed in domestic animals and wildlife with greater interest (*3*).

Emerging diseases in animals, especially farm animals, involve economic losses through direct (deaths, culls, movement restriction, laboratory tests) and indirect (decreased consumption of animal products, tourism decline) costs. For example, the cost of the BSE epidemic in the United Kingdom has been high, both for control measures and through lost trade, >£740 million in 1997 alone (http://www.defra.gov.uk/animalh/bse/general/qa/section9.html, accessed 9 May 2005). In addition, BSE has been implicated in the deaths of 150 persons in the United Kingdom to date (http://www.cjd.ed.ac.uk/figures.htm, accessed 9 May 2005). In 1997 and 2004, outbreaks of avian influenza A (H5N1) in Asia, with transmission to humans, led to massive destruction of poultry to avert a pandemic (*4*).

Because diseases will continue to emerge, the potential unexpected or atypical features of future health problems makes surveillance particularly challenging (*5*). No single data source captures all the information required for surveillance. Early clinical detection is one of the cornerstones (*6*) regarding unexpected diseases insofar as the surveillance activities of the veterinarians can be focused and systematized. This article presents approaches and tools focused on detecting potentially emerging diseases in farm animals through 3 information systems being tested in New Zealand, the United States, and France.

## Approaches To Detect Clinical Emerging Issues

Most surveillance programs deal with a restricted set of known diseases that fail to address the challenges of looking for the unknown. However, in the United States, many new human infectious diseases have been recognized by examining illnesses without identified cause (*7*). Furthermore, in Great Britain, the unusual neurologic clinical signs in cattle forewarned of a new disease, BSE (*8*). Developing the ability to detect atypical syndromes in a timely fashion is critical to reducing the impact of disease emergence.

Programs targeted to detect atypical animal diseases follow 2 approaches. The first approach, syndromic surveillance, monitors disease trends by grouping clinical diseases into syndromes on the basis of clinical features rather than specific diagnoses (*9*). Even though syndromic surveillance systems seek to minimize the amount of data collected from each case, their main drawback is the heavy reporting load and requirement for disciplined reporting of recognized case data.

The second approach focuses on detecting individual atypical cases. Based on how previous emerging diseases have been detected (Table 1), atypical cases can arise from a new disease that shows clinical signs the clinician cannot link to a known disease. Alternatively, they arise from a known disease expressed atypically through unusual clinical signs, atypical region or species, or increased severity. An atypical case can also result from the detection of a rare or inadequately documented sporadic disease. Detection focused on atypical cases requires a lighter reporting load than syndromic surveillance, but the practitioner response is likely to be variable and require regular prompting.

**Table 1:** Examples of emerging diseases and how they were detected and identified in farm animals in the last 20 years

Emerging disease (etiology)	Species	Location, date	Detection keys at time of emergence	Ref.
Blue tongue (*Reoviridae*)	Sheep	Mediterranean basin, 1998–01	Disease normally occurring south of the Mediterranean basin	(*10*)
Border (*Flaviviridae*)	Sheep	France, 1994	Unusual death rates and clinical signs for the region: abortion, nervous signs, hydrocephalus	(*11*)
Bovine leukocyte adhesion deficiency (CD 18 gene mutation)	Holstein cattle	Different countries, 1980s	Unusual death rates in calves with recurrent infections	(*12*)
Bovine spongiform encephalopathy (prion)	Cattle	Great Britain, 1980	Unusual clinical and pathologic signs in the species: progressive neurologic disorders, gray matter vacuolation and scrapie associated fibrils	(*8*)
Complex vertebral malformation (SLC35A3 gene mutation)	Dairy cattle	Denmark, 2000	Unknown lethal congenital defect	(*13*)
Epizootic rabbit enteropathy (unidentified virus)	Rabbits	Europe, 1996	Unknown disease: serious enteritis, highly contagious, often fatal	(*14*)
Hendra virus disease (Paramyxovirus)	Horses, humans	Australia, Papua New Guinea, 1994	Sudden outbreak of acute respiratory syndrome in horses	(*15*)
Highly pathogenic avian influenza (H5N1 virus)	Poultry, humans	Southeast Asian countries, 2003–2004	Outbreak of highly pathogenic avian influenza in poultry	(*16*)
Nipah virus disease (Paramyxovirus)	Swine, humans	Malaysia and Singapore, 1998	Outbreak of unknown highly contagious disease in pigs: acute fever, respiratory signs, neurologic signs; encephalitis in humans	(*17*)
Porcine dermatitis and nephropathy syndrome (suspected porcine circovirus 2)	Swine	United Kingdom, 1993	Unusual clinical signs: unusual skin lesions in patches and plaques	(*18*)
Porcine reproductive and respiratory syndrome (*Arteriviridae*)	Swine	North America, 1987	Unusual association of: swine infertility, respiratory problems, abortion, and cyanotic ears	(*19*)
Post-weaning multisystemic wasting syndrome (Suspected porcine circovirus 2)	Swine	Canada, 1990	Unusual association of: wasting, dyspnea, enlarged lymph nodes, diarrhea, pallor, and jaundice	(*18*)
Rabbit hemorrhagic disease (*Caliciviridae*)	Rabbits	China, 1984	Unusual high death rate and hemorrhage	(*20*)
West Nile fever (*Flaviviridae*)	Humans, crows	United States, 1999	Unusual cluster of human encephalitis, extensive death rate in crows, deaths of exotic birds in a zoo	(*21*)

## Information Systems To Analyze Clinical Data from Farm Animals

Advances in information technology have allowed novel uses of Web and pocket personal computer applications, which provide speed, efficiency, interactivity, and security. In 1997 in Colorado, veterinarians provided information regarding unusual clinical events through the Internet (*22*); however, the program was discontinued because of poor user response. Subsequent approaches and tools to clinically detect potential emerging diseases in farm animals are presented here through 3 prototype information systems: the Veterinary Practitioner Aided Disease Surveillance System (VetPAD, New-Zealand) (*23*), which is in its third year with 7 pilot veterinarians; the Rapid Syndrome Validation Project—Animal (RSVP-A, USA) (*24*), which has been piloted among 17 veterinarians in Kansas since 2003 and 10 veterinarians in New Mexico since 2005; and the "émergences" system (available from http://www.inra.fr/maladies-emergentes) (*25*), which was pilot tested with 12 veterinarians in 2003 and has been pilot tested with 30 veterinarians since September 2005 (Table 2). All systems are being tested in cattle because veterinary practitioners have high rates of on-farm contact with bovine herds.

**Table 2:** Comparison of 3 information systems to analyze animal disease through clinical observations*

		VetPAD	RSVP-A	"émergences"
General information				
		Country of origin	New Zealand	United States	France (available in French, Spanish, English)
		Species targeted/ where applied	Farm animals/dairy cattle	Cattle/cattle	Domestic animals/cattle
		Means of recording	Pocket PC	Palm device, PC with Internet, wireless microbrowser	PC with Internet, cell phone
		Pilot tests	7 veterinarians in New Zealand, 2004–2005	1) 17 veterinarians in Kansas, 2003–2006; 2) 10 veterinarians in New Mexico, 2005–2006	1) 12 veterinarians in France, 2003; 2) 30 veterinarians in 2 French counties, 2005–2007
Record				
		Type of clinical data	Syndromic surveillance: all clinical cases	Syndromic surveillance: 6 syndromes (see text)	Atypical syndromes and customized targeted diseases, record of the absence of cases
		Main epidemiologic data	Farm localization and ownership, number affected, dead, and at risk	Type of farm, production stage, localization, number affected, dead, and at risk	Type of farm, production stage, localization, contact with other animals, number affected, dead, and at risk
		Main data related to the disease	Clinical syndrome/specific clinical diagnosis	Type of syndrome, some additional clinical observation	Reasons for notification, main clinical characteristics
		Type of data field	Pick-up lists, check boxes, free text fields	Pick-up lists, check boxes, free text fields	Pick-up lists, check boxes, free text fields
		Other record	Photos		Photos, epidemiologic questionnaires
Output				
		Related to epidemiologic surveillance	Analysis and reporting at the practitioner, regional, and national levels	Incident pattern reports from coverage areas defined by practitioners, maps	Practice statistics, statistics with all reported cases, access to all reports
		Other outputs	Visit management, list of remedies, printouts for clients (wireless technology)		
Further technical developments				
			GPS capability, linkage of clinical to laboratory diagnosis, barcode scanning	GPS capability	Implementation of anatomo-pathology and laboratory analyses

*VetPAD, Veterinary Practitioner Aided Disease; RSVP-A, Rapid Syndrome Validation Project – Animal; "émergences," information system in France; PC, personal computer; GPS, global positioning system.

### Data Capture and Strategies

All 3 systems work from the premise that practicing veterinarians hold key animal health information, which could improve means for early detection of emerging disease if aggregated efficiently through advanced information technology. While all systems capture basic epidemiologic data, they each represent a different approach to emerging disease surveillance.

VetPAD has a syndromic surveillance approach. It can include all farm animals. It collects data describing every case. Cases are categorized by using dropdown lists, check boxes, and a clinical diagnosis. Based on the categorizations, cases can be flexibly aggregated for syndromic surveillance. The strategy to minimize the surveillance reporting impact is to provide a tool capturing the ordinary business data veterinarians must manage anyway (medical records, inventory, and accounts). Surveillance data are a subset of these other data.

The RSVP-A system employs an aggregation-based syndromic surveillance but focuses on a restricted set of syndromes (nonneonatal diarrhea, neurologic dysfunction or recumbency, abortion or birth defect, unexpected death, erosive or ulcerative lesions, and unexplained feed refusal or weight loss). These syndromes are defined to cover clinical signs of emerging disease other than the common production problems on which most livestock enterprises are focused. Practitioners determine the specific syndrome each case best fits and record demographic data about the diseased animals. The RSVP-A system also requests additional clinical observations potentially useful to further characterize incident patterns. The strategy to minimize the reporting impact is to focus on less common clinical syndromes and to make data capture for each case require <1 minute.

"émergences" has a different approach as it targets atypical cases and specific diseases, which correspond to known diseases hypothesized to be emerging. Forms are available (see an example of atypical case form, Figure) for reporting epidemiologic and clinical data. The system requests a follow-up description of each case's evolution and monthly confirmations of vigilance from veterinarians reporting no cases. Moreover, atypical cases can be categorized by the system administrator according to clinical description similarities to facilitate exploration of their potential links. The system has generic features, making it available for any country, any disease, and any domestic species. Description of atypical cases for "émergences" is a less frequent and more open process than the syndromic surveillance methods.

**Figure:**
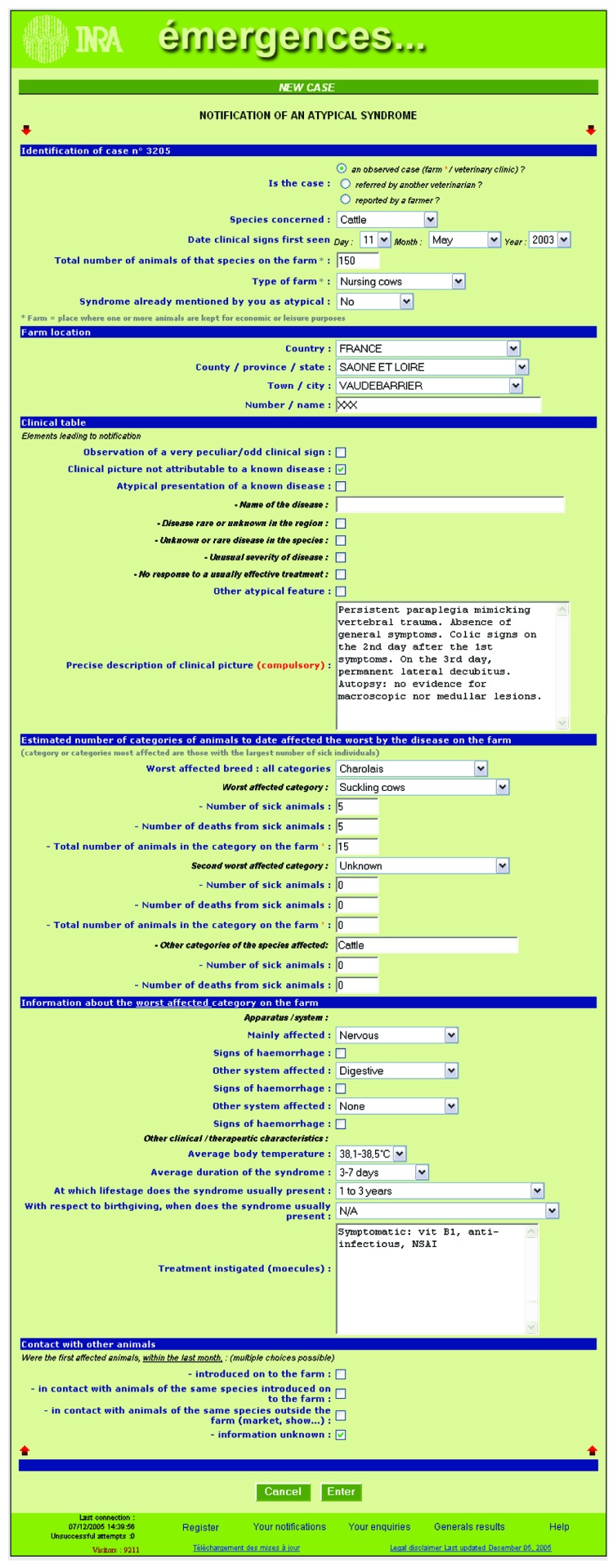
Sample of online form reporting epidemiologic and clinical data.

In all these systems, routine data recording is simplified by the use of pick-up lists. However, free text fields are also available, as the unexpected often does not fit in predefined fields. VetPAD and RSVP-A use mobile telephones or personal data assistants for data capture. "émergences" primarily uses the Internet.

### Output and Statistics

A successful surveillance system must be able to keep veterinarians engaged and continuing to submit data after the novelty of the new system wears off. Systems can provide value to a veterinarian with useful management tools, which are available in VetPAD, and by enhancing their clinical expertise and intellectual curiosity. To trigger interactions and learning from participants' experiences, practitioners participating in "émergences" have access to all case descriptions. In addition, illness and death rates are available in real time either at the clientele level ("émergences") or at a custom-made level ("émergences," RSVP-A). In VetPAD, customized reports are available to involved parties.

One output of these surveillance systems is an indication of unusual events that require additional investigation. This investigation might include communication with other veterinarians to find additional cases, targeted epidemiologic studies, research projects, or control programs.

Other outputs are data upon which analyses can be conducted. A challenge is the categorization of reports to identify possible etiologic links. Procedures based on contextual analysis must be developed to analyze pick-up list data as well as free text (*26*). Each system must also address the challenge of detecting increased incidence of a rare event. Two types of situations can be considered. The first is the emergence from a "zero case" situation (e.g., BSE occurred probably as erratic cases before its amplification [27]). Incidence threshold analysis needed for this situation requires methods such as the evaluation of record process (*28*). Moreover, the constructed statistics should be robust with a small number of cases and allow differentiation of sporadic cases from emergence (*29*). The second situation is the emergence of clusters of highly pathogenic variants of an endemic disease. Spatial-temporal analysis can provide helpful insights concerning baseline patterns of clinical syndromes and aberrations from them, which can trigger further investigation.

## Limitations and Evaluation of Systems Based on Clinical Observation

### Limitations

Atypical case detection is limited by practitioners' experience, knowledge, vigilance, and willingness to report findings (*30*). Multiple, similar reports of atypical cases improve confidence that a new disease is emerging. Making case data available through surveillance systems, such as the 3 we have indicated, will also foster basic common knowledge and shared practical experience among veterinarians. Because surveillance for the unknown requires a mindset different from surveillance of the known, notification quality and vigilance should be enhanced by specific training courses (*31*).

A substantial limitation of syndromic surveillance is the need to establish baseline levels for defined syndromes. This step requires time and resources; however, without them, we cannot know when the incidence of a syndrome has significantly increased. VetPAD and RSVP-A are developing such baselines.

Economic consideration leaves few alternatives to clinical detection of farm animal diseases. Laboratory analyses are infrequently performed and generally more basic compared to human medicine (*32*). However, slaughterhouses and other assembly points do provide surveillance opportunities.

Finally, a clinical reporting tool alone is only the first step to determine if the cases share an etiologic pathway. Review by expert clinicians, necropsy findings, immunologic screenings, and focused epidemiologic studies play key roles in such determination (*33*). Similarities between distinct submitted atypical cases provide additional evidence. For example, BSE was identified as a novel syndrome through epidemiologic, clinical, and pathologic findings (*8*).

### Evaluation

To determine whether to extend an information system, several points must be reviewed. First, the activity and number of participating veterinarians can be evaluated by quantifying indicators such as number of entries submitted, number of atypical cases entered, and participants' levels of accessing posted results. Moreover, all systems include reference diseases or symptoms for which descriptive statistics are available, which can serve to check quality recording (e.g., babesiosis in the "émergences" pilot study). In addition, the likelihood of detecting an emerging event is high. Many rare diseases are not defined in cattle, so a dedicated information system should detect >1 unexpected event over the test period. For example, the initial "émergences" pilot found 3 sets of clinical signs not linked to a known disease (persistent, ultimately fatal paraplegia, without general clinical signs [Figure]; weight loss, depilation at the extremities leading to death; and congenital cataract neither linked to bovine virus diarrhea nor familial history) and 1 rare known syndrome (facial eczema). Finally, the decision to extend a detection system will depend largely on the interest veterinarians hold and on the inclusion of new diseases as a national surveillance objective (*6**,**34*).

## Other Systems To Capture Clinical Data

We have presented examples of clinical data capture from cattle herds at the veterinary level, in which sufficient individual health data are available. For species concerned by herd health approaches (sheep, poultry), initiatives have been taken for information systems through online questionnaires answered by farmers (*35*). In 1 such system, New Zealand producers must complete questionnaires targeted on diseases that occurred in the previous 12 months and have clinical signs similar to exotic diseases. The ultimate research goal is to develop a disease sentinel Web module to integrate with veterinary practice Web sites. The main problem is the disparity in response quality between farmers.

The reality of an emergence can be tested by survey of a set of representative herds. In the United States, the National Animal Health Monitoring System is not designed to collect information regarding emerging diseases per se; however, questions about a previously identified emerging disease have been inserted into surveys. In addition, the National Animal Health Monitoring System has provided baseline data on emerging disease analysis and assessment. In France, the Central Service for Survey and Statistical Studies, which runs economic surveys among a representative national sample of herds, has added specific questions regarding animal health issues (*36*).

In addition to farm animals, pets, zoo animals, and wildlife must be considered as sources of transmission and reservoirs for emerging diseases. For pets and zoo animals, tools similar to the ones proposed can be adapted because these animals are regularly seen by veterinarians. Wildlife can be a source of new farm animal or human diseases and is affected by many farm animal diseases (Table 1). Thus, all observations of health problems in wildlife can potentially contribute relevant information for human or domestic animal populations (*37*). However, the ability to closely monitor clinical signs is lacking. Death rate is the most feasible way to monitor wildlife health and has indeed been the detection trigger of many emerging diseases (*38*). Testing sampled healthy animals for a set of diseases is another strategy, but few disease surveillance programs not targeted at specific diseases are in place (e.g., "marine mammal strandings" project in United Kingdom [39]). One of the key challenges remains to bring professional and amateur outdoorsmen to report wildlife health observations through an information system flexible enough to encompass all species and situations. New forms dedicated to wildlife with appropriate location (instead of client or farm) could be added to the information systems already adapted to several species (VetPAD and "émergences"). Alternatives such as monitoring risk factors for emergence (e.g., encroachment of habitats), as well as minimizing contact between domestic and wild species by good, on-farm biosecurity, could reduce the likelihood of new domestic animal or human diseases emerging from wildlife reservoirs. In all cases, approaches must seek to increase collaboration among wildlife and domestic animals health workers to break down traditional boundaries between fields.

## Conclusion and Interest for Human Health

Much effort is being put into developing new tools to detect emerging diseases through veterinary practitioners. If successful, this effort will also define the "normal" clinical baseline for syndromes and rare diseases, allowing statistical confirmation that an atypical syndrome is emerging. In addition to building new information technologies, early disease identification with timely responses requires synergy across a group of partners, including those who traditionally interact in animal health management as well as in public health (*40*) and across geopolitical boundaries. Although human and animal worlds remain fairly separated, initiatives are narrowing this separation. For instance, integration of emerging animal disease surveillance systems with those in the human arena is proposed in the UK's "RADAR" veterinary surveillance information management system (*41*). Furthermore, during the "émergences" test phase, the Health National Institute agreed to cooperate in the event an animal issue with potential public health implications was identified. Finally, the most relevant challenge is to promote joint human-animal projects concerning potentially common emerging diseases, such as the avian-porcine-human influenza complex. Effective combination of such emerging disease surveillance systems would result in earlier identification of potential issues, providing opportunity for quicker response.
