# Correction, Vol. 12, No. 2

**DOI:** 10.3201/eid1204.C11204

**Published:** 2006-04

**Keywords:** errata, erratum, correction

In "Detecting Emerging Diseases in Farm Animals through Clinical Observations" by G. Vourc'h et al., acknowledgment of contributions by Sandia National Laboratories, New Mexico State University, New Mexico Department of Agriculture, and Kansas State University were omitted. Acknowledgments should include the following:

Sandia National Laboratories designed and developed the original RSVP surveillance system, a system with applications in both human and animal disease surveillance.

Sandia National Laboratories and New Mexico State University/New Mexico Department of Agriculture are primary collaborators, along with Kansas State University, on the RSVP-A project that has been jointly pursued since 2003. The opinions on RSVP-A in this article do not necessary reflect all of the project's collaborating parties.

The corrected article appears online at http://wwwnc.cdc.gov/eid/article/12/2/05-0498_article.htm

We regret the omission and any confusion it may have caused.

